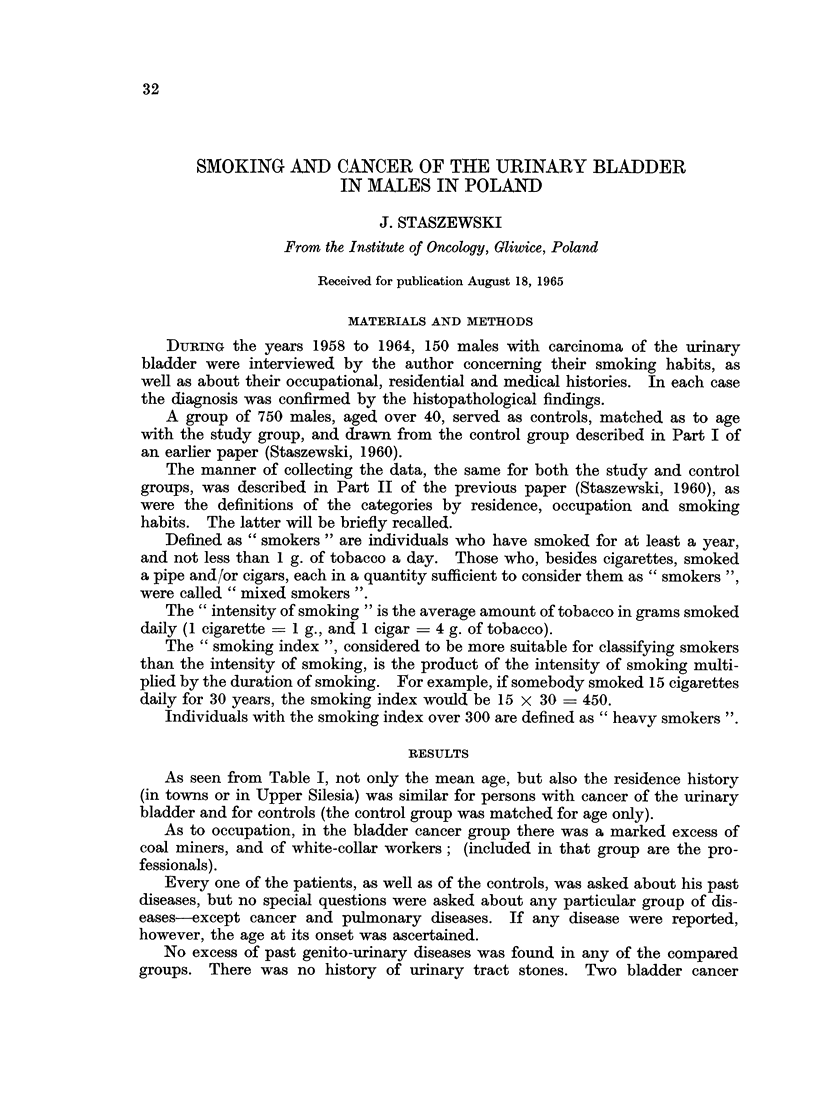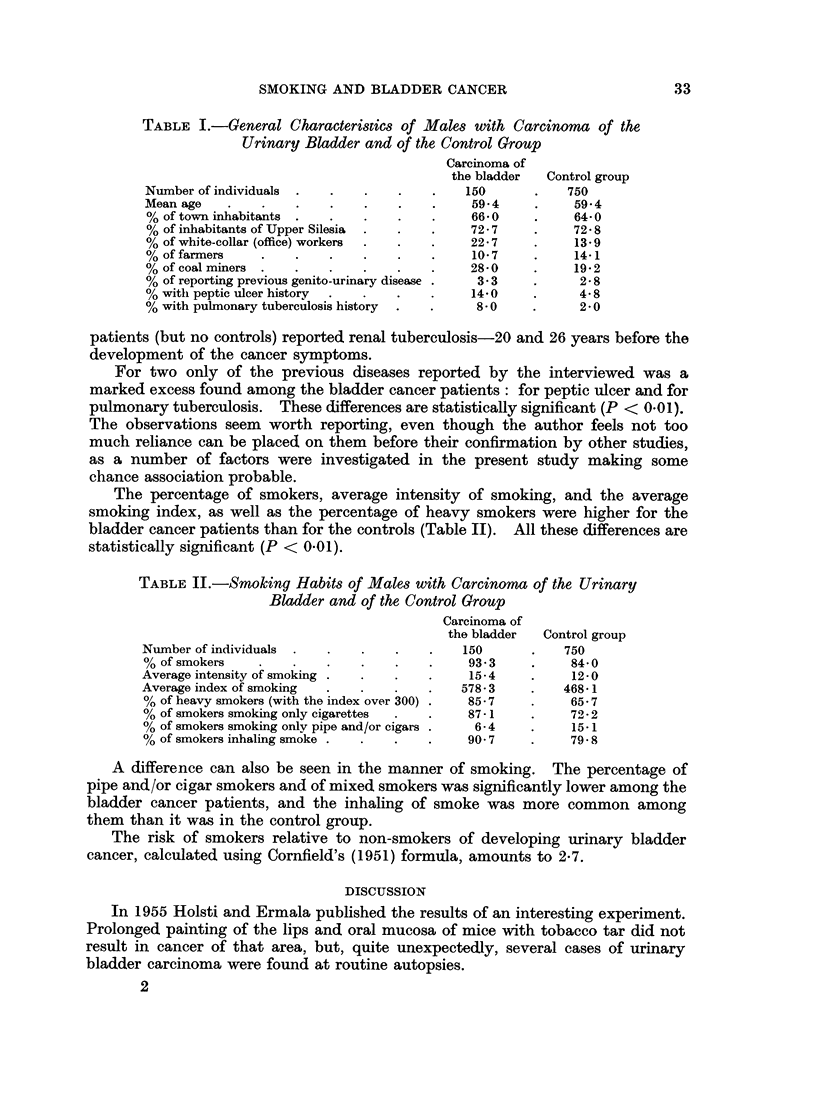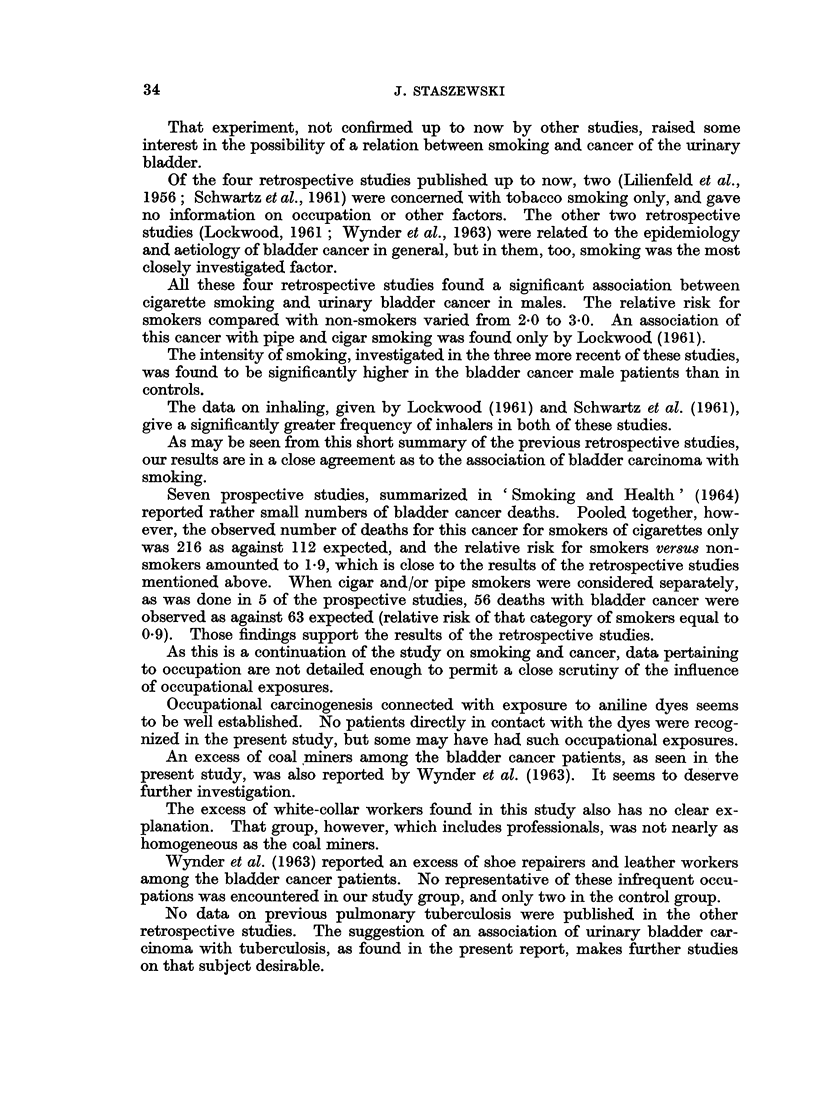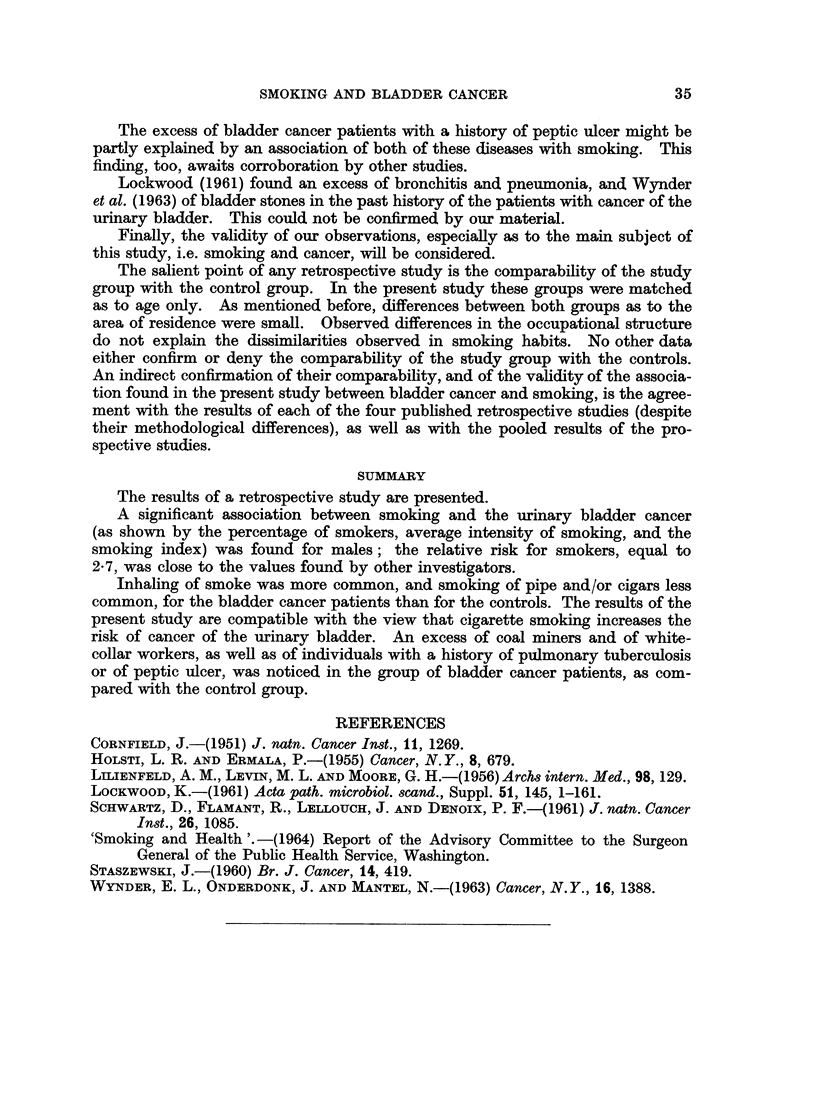# Smoking and cancer of the urinary bladder in males in Poland.

**DOI:** 10.1038/bjc.1966.4

**Published:** 1966-03

**Authors:** J. Staszewski


### SMOKING AND CANCER OF THE URINARY BLADDER IN MALES IN POLAND

J. STASZEWSKI

*From the Institute of Oncology, Gliwice, Poland*

Received for publication August 18, 1965

### MATERIALS AND METHODS

DURING the years 1958 to 1964, 150 males with carcinoma of the urinary bladder were interviewed by the author concerning their smoking habits, as well as about their occupational, residential and medical histories. In each case the diagnosis was confirmed by the histopathological findings.

A group of 750 males, aged over 40, served as controls, matched as to age with the study group, and drawn from the control group described in Part I of an earlier paper (Staszewski, 1960).

The manner of collecting the data, the same for both the study and control groups, was described in Part II of the previous paper (Staszewski, 1960), as were the definitions of the categories by residence, occupation and smoking habits. The latter will be briefly recalled.

Defined as " smokers " are individuals who have smoked for at least a year, and not less than 1 g. of tobacco a day. Those who, besides cigarettes, smoked a pipe and/or cigars, each in a quantity sufficient to consider them as " smokers ", were called " mixed smokers ".

The " intensity of smoking " is the average amount of tobacco in grams smoked daily (1 cigarette = 1 g., and 1 cigar = 4 g. of tobacco).

The " smoking index ", considered to be more suitable for classifying smokers than the intensity of smoking, is the product of the intensity of smoking multiplied by the duration of smoking. For example, if somebody smoked 15 cigarettes daily for 30 years, the smoking index would be $15 \times 30 = 450$.

Individuals with the smoking index over 300 are defined as " heavy smokers ".

### RESULTS

As seen from Table I, not only the mean age, but also the residence history (in towns or in Upper Silesia) was similar for persons with cancer of the urinary bladder and for controls (the control group was matched for age only).

As to occupation, in the bladder cancer group there was a marked excess of coal miners, and of white-collar workers ; (included in that group are the professionals).

Every one of the patients, as well as of the controls, was asked about his past diseases, but no special questions were asked about any particular group of diseases—except cancer and pulmonary diseases. If any disease were reported, however, the age at its onset was ascertained.

No excess of past genito-urinary diseases was found in any of the compared groups. There was no history of urinary tract stones. Two bladder cancer

TABLE I.—*General Characteristics of Males with Carcinoma of the Urinary Bladder and of the Control Group*

| | Carcinoma of the bladder | Control group |
|---|---|---|
| Number of individuals | 150 | 750 |
| Mean age | 59·4 | 59·4 |
| % of town inhabitants | 66·0 | 64·0 |
| % of inhabitants of Upper Silesia | 72·7 | 72·8 |
| % of white-collar (office) workers | 22·7 | 13·9 |
| % of farmers | 10·7 | 14·1 |
| % of coal miners | 28·0 | 19·2 |
| % of reporting previous genito-urinary disease | 3·3 | 2·8 |
| % with peptic ulcer history | 14·0 | 4·8 |
| % with pulmonary tuberculosis history | 8·0 | 2·0 |

patients (but no controls) reported renal tuberculosis—20 and 26 years before the development of the cancer symptoms.

For two only of the previous diseases reported by the interviewed was a marked excess found among the bladder cancer patients : for peptic ulcer and for pulmonary tuberculosis. These differences are statistically significant ($P < 0{\cdot}01$). The observations seem worth reporting, even though the author feels not too much reliance can be placed on them before their confirmation by other studies, as a number of factors were investigated in the present study making some chance association probable.

The percentage of smokers, average intensity of smoking, and the average smoking index, as well as the percentage of heavy smokers were higher for the bladder cancer patients than for the controls (Table II). All these differences are statistically significant ($P < 0{\cdot}01$).

TABLE II.—*Smoking Habits of Males with Carcinoma of the Urinary Bladder and of the Control Group*

| | Carcinoma of the bladder | Control group |
|---|---|---|
| Number of individuals | 150 | 750 |
| % of smokers | 93·3 | 84·0 |
| Average intensity of smoking | 15·4 | 12·0 |
| Average index of smoking | 578·3 | 468·1 |
| % of heavy smokers (with the index over 300) | 85·7 | 65·7 |
| % of smokers smoking only cigarettes | 87·1 | 72·2 |
| % of smokers smoking only pipe and/or cigars | 6·4 | 15·1 |
| % of smokers inhaling smoke | 90·7 | 79·8 |

A difference can also be seen in the manner of smoking. The percentage of pipe and/or cigar smokers and of mixed smokers was significantly lower among the bladder cancer patients, and the inhaling of smoke was more common among them than it was in the control group.

The risk of smokers relative to non-smokers of developing urinary bladder cancer, calculated using Cornfield's (1951) formula, amounts to 2·7.

### DISCUSSION

In 1955 Holsti and Ermala published the results of an interesting experiment. Prolonged painting of the lips and oral mucosa of mice with tobacco tar did not result in cancer of that area, but, quite unexpectedly, several cases of urinary bladder carcinoma were found at routine autopsies.

That experiment, not confirmed up to now by other studies, raised some interest in the possibility of a relation between smoking and cancer of the urinary bladder.

Of the four retrospective studies published up to now, two (Lilienfeld *et al.*, 1956 ; Schwartz *et al.*, 1961) were concerned with tobacco smoking only, and gave no information on occupation or other factors. The other two retrospective studies (Lockwood, 1961 ; Wynder *et al.*, 1963) were related to the epidemiology and aetiology of bladder cancer in general, but in them, too, smoking was the most closely investigated factor.

All these four retrospective studies found a significant association between cigarette smoking and urinary bladder cancer in males. The relative risk for smokers compared with non-smokers varied from 2·0 to 3·0. An association of this cancer with pipe and cigar smoking was found only by Lockwood (1961).

The intensity of smoking, investigated in the three more recent of these studies, was found to be significantly higher in the bladder cancer male patients than in controls.

The data on inhaling, given by Lockwood (1961) and Schwartz *et al.* (1961), give a significantly greater frequency of inhalers in both of these studies.

As may be seen from this short summary of the previous retrospective studies, our results are in a close agreement as to the association of bladder carcinoma with smoking.

Seven prospective studies, summarized in ‘ Smoking and Health ’ (1964) reported rather small numbers of bladder cancer deaths. Pooled together, however, the observed number of deaths for this cancer for smokers of cigarettes only was 216 as against 112 expected, and the relative risk for smokers *versus* non-smokers amounted to 1·9, which is close to the results of the retrospective studies mentioned above. When cigar and/or pipe smokers were considered separately, as was done in 5 of the prospective studies, 56 deaths with bladder cancer were observed as against 63 expected (relative risk of that category of smokers equal to 0·9). Those findings support the results of the retrospective studies.

As this is a continuation of the study on smoking and cancer, data pertaining to occupation are not detailed enough to permit a close scrutiny of the influence of occupational exposures.

Occupational carcinogenesis connected with exposure to aniline dyes seems to be well established. No patients directly in contact with the dyes were recognized in the present study, but some may have had such occupational exposures.

An excess of coal miners among the bladder cancer patients, as seen in the present study, was also reported by Wynder *et al.* (1963). It seems to deserve further investigation.

The excess of white-collar workers found in this study also has no clear explanation. That group, however, which includes professionals, was not nearly as homogeneous as the coal miners.

Wynder *et al.* (1963) reported an excess of shoe repairers and leather workers among the bladder cancer patients. No representative of these infrequent occupations was encountered in our study group, and only two in the control group.

No data on previous pulmonary tuberculosis were published in the other retrospective studies. The suggestion of an association of urinary bladder carcinoma with tuberculosis, as found in the present report, makes further studies on that subject desirable.

The excess of bladder cancer patients with a history of peptic ulcer might be partly explained by an association of both of these diseases with smoking. This finding, too, awaits corroboration by other studies.

Lockwood (1961) found an excess of bronchitis and pneumonia, and Wynder *et al.* (1963) of bladder stones in the past history of the patients with cancer of the urinary bladder. This could not be confirmed by our material.

Finally, the validity of our observations, especially as to the main subject of this study, i.e. smoking and cancer, will be considered.

The salient point of any retrospective study is the comparability of the study group with the control group. In the present study these groups were matched as to age only. As mentioned before, differences between both groups as to the area of residence were small. Observed differences in the occupational structure do not explain the dissimilarities observed in smoking habits. No other data either confirm or deny the comparability of the study group with the controls. An indirect confirmation of their comparability, and of the validity of the association found in the present study between bladder cancer and smoking, is the agreement with the results of each of the four published retrospective studies (despite their methodological differences), as well as with the pooled results of the prospective studies.

### SUMMARY

The results of a retrospective study are presented.

A significant association between smoking and the urinary bladder cancer (as shown by the percentage of smokers, average intensity of smoking, and the smoking index) was found for males; the relative risk for smokers, equal to 2·7, was close to the values found by other investigators.

Inhaling of smoke was more common, and smoking of pipe and/or cigars less common, for the bladder cancer patients than for the controls. The results of the present study are compatible with the view that cigarette smoking increases the risk of cancer of the urinary bladder. An excess of coal miners and of white-collar workers, as well as of individuals with a history of pulmonary tuberculosis or of peptic ulcer, was noticed in the group of bladder cancer patients, as compared with the control group.

### REFERENCES

CORNFIELD, J.—(1951) *J. natn. Cancer Inst.*, **11**, 1269.

HOLSTI, L. R. AND ERMALA, P.—(1955) *Cancer, N.Y.*, **8**, 679.

LILIENFELD, A. M., LEVIN, M. L. AND MOORE, G. H.—(1956) *Archs intern. Med.*, **98**, 129.

LOCKWOOD, K.—(1961) *Acta path. microbiol. scand.*, Suppl. **51**, 145, 1–161.

SCHWARTZ, D., FLAMANT, R., LELLOUCH, J. AND DENOIX, P. F.—(1961) *J. natn. Cancer Inst.*, **26**, 1085.

'Smoking and Health'.—(1964) Report of the Advisory Committee to the Surgeon General of the Public Health Service, Washington.

STASZEWSKI, J.—(1960) *Br. J. Cancer*, **14**, 419.

WYNDER, E. L., ONDERDONK, J. AND MANTEL, N.—(1963) *Cancer, N.Y.*, **16**, 1388.